# Perfect X-ray focusing via fitting corrective glasses to aberrated optics

**DOI:** 10.1038/ncomms14623

**Published:** 2017-03-01

**Authors:** Frank Seiboth, Andreas Schropp, Maria Scholz, Felix Wittwer, Christian Rödel, Martin Wünsche, Tobias Ullsperger, Stefan Nolte, Jussi Rahomäki, Karolis Parfeniukas, Stylianos Giakoumidis, Ulrich Vogt, Ulrich Wagner, Christoph Rau, Ulrike Boesenberg, Jan Garrevoet, Gerald Falkenberg, Eric C. Galtier, Hae Ja Lee, Bob Nagler, Christian G. Schroer

**Affiliations:** 1 Institute of Structural Physics, Technische Universität Dresden, 01062 Dresden, Germany; 2 Deutsches Elektronen-Synchrotron DESY, Notkestraße 85, 22607 Hamburg, Germany; 3 Institute of Optics and Quantum Electronics, Friedrich-Schiller-Universität Jena, Max-Wien-Platz 1, 07743 Jena, Germany; 4 Linac Coherent Light Source, SLAC National Accelerator Laboratory, 2575 Sand Hill Road, Menlo Park, California 94025, USA; 5 Institute of Applied Physics, Friedrich-Schiller-Universität Jena, Albert-Einstein-Straße 15, 07745 Jena, Germany; 6 KTH Royal Institute of Technology, Biomedical and X-ray Physics, Albanova University Center, 106 91 Stockholm, Sweden; 7 Diamond Light Source Ltd, Diamond House, Harwell Science and Innovation Campus, Didcot, Oxfordshire OX11 0DE, UK; 8 Department Physik, Universität Hamburg, Luruper Chaussee 149, 22761 Hamburg, Germany

## Abstract

Due to their short wavelength, X-rays can in principle be focused down to a few nanometres and below. At the same time, it is this short wavelength that puts stringent requirements on X-ray optics and their metrology. Both are limited by today's technology. In this work, we present accurate at wavelength measurements of residual aberrations of a refractive X-ray lens using ptychography to manufacture a corrective phase plate. Together with the fitted phase plate the optics shows diffraction-limited performance, generating a nearly Gaussian beam profile with a Strehl ratio above 0.8. This scheme can be applied to any other focusing optics, thus solving the X-ray optical problem at synchrotron radiation sources and X-ray free-electron lasers.

## Abstract (web summary)

X-ray optics are notoriously challenging to fabricate due to the strict tolerances that result from the short wavelength of radiation. Here, Seiboth *et al*. carefully quantify aberrations in complex X-ray lenses and correct them with an easy-to-fabricate broadband phase plate.

Modern synchrotron radiation sources and X-ray free-electron lasers (XFELs) provide highly brilliant X-ray beams that allow studying the structure and dynamics of matter from atomic distances and a few femtoseconds to macroscopic dimensions and seconds. Creating small and intense X-ray beams is crucial to confine the beam and concentrate the radiation onto the sample. Ideally, this would require diffraction-limited X-ray optics with high numerical aperture (NA) that are at the same time stable in the intense XFEL pulses^1^. So far, mainly mirror systems^2^,^3^ and beryllium compound refractive lenses^4^,^5^ (Be CRLs) are in routine operation at XFELs.

Due to the short wavelength of X-rays, the fabrication of X-ray optics requires the most advanced technologies, such as lithographic nanofabrication for diffractive^6^ and refractive optics^7^, surface figuring with atomic precision for total reflection and multilayer mirrors^8^,^9^ and thin-film technologies for multilayer optics^10^. Today, most X-ray optics are limited by fabrication limitations, and tradeoffs need to be made in terms of aberration-free performance and highest possible NA.

To characterize the optics and the resulting nanobeams, metrology has become more and more demanding. Over the past years, X-ray scanning coherent diffraction microscopy, also known as ptychography^11^, has evolved to one of the most important methods for X-ray beam and optics characterization^12^. It can be used to quantitatively retrieve the complex wavefield of a focused X-ray beam and, hence, reveals wavefront errors with unprecedented sensitivity and spatial resolution^13^,^14^. From the three-dimensional reconstruction of the focal spot volume^4^, detailed information about aberrations of the optical system can be deduced^15^,^16^.

Here, we present a general scheme to assess aberrations of an X-ray optical system under working conditions and correct them by introducing an appropriate X-ray phase plate into the optical path to achieve diffraction-limited focusing. The phase plate operates in transmission and is based on refraction. Hence, it is largely insensitive to small shape and surface inaccuracies of a few μm and can correct residual aberrations originating from surface errors of reflective optics, zone deformations in diffractive optics and accumulated surface errors in larger refractive lens stacks. Using ptychography, we measured the complex wavefield of a focused X-ray beam generated by Be CRLs at the Linac Coherent Light Source (LCLS). The detailed knowledge of the complex wavefield was used to fabricate a corrective phase plate that compensates for the aberrations and thus creates a diffraction-limited focus when introduced into the beam following the lens. The method can be applied very generally to solve the X-ray focusing problem at synchrotron radiation sources and XFELs and will affect fields as diverse as X-ray microscopy and high-resolution imaging^17^,^18^, serial crystallography^19^,^20^, creating matter in extreme conditions^21^, nonlinear X-ray optics^22^ and single-molecule imaging^23^.

## Results

### Initial aberration analysis

**Figure 1:**
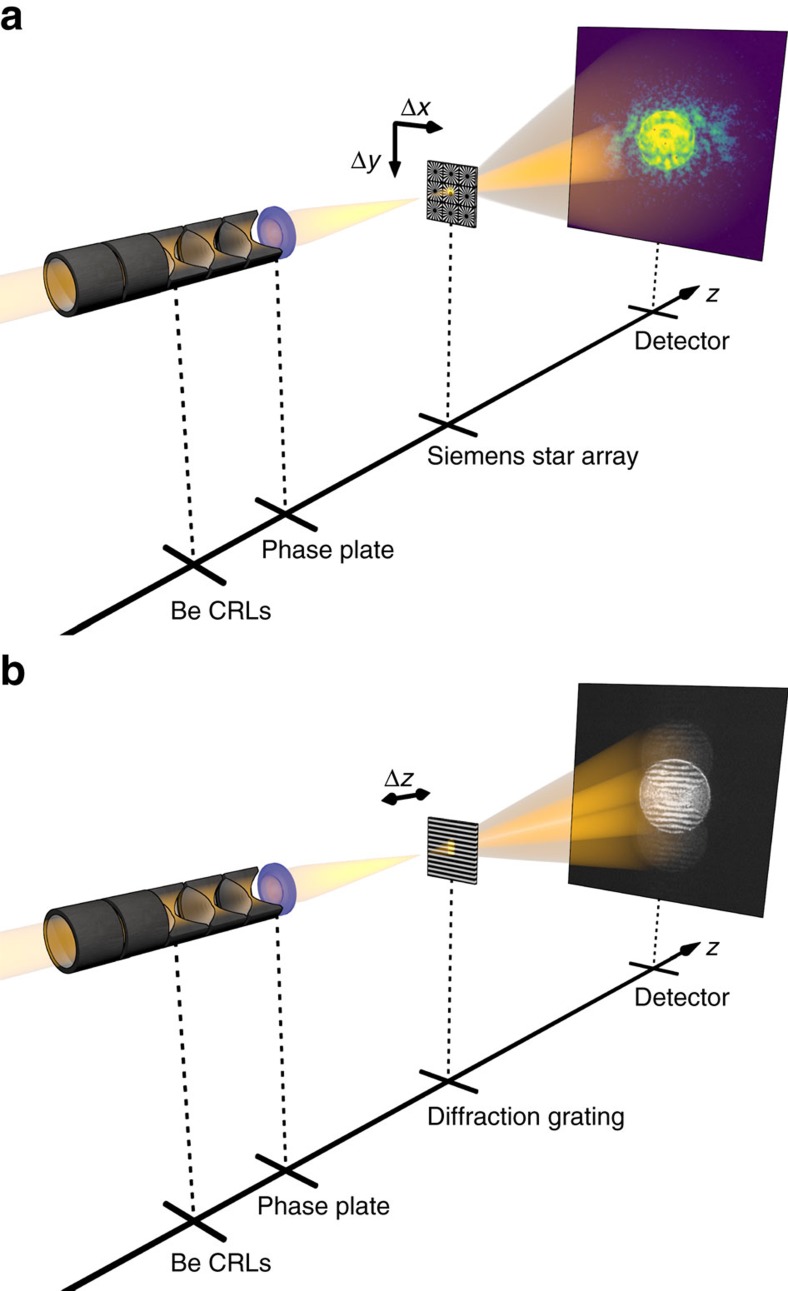
Experimental setup. X-rays with *E*=8.2 keV (selected by a Si-111 monochromator) were focused by a compound refractive lens made of beryllium (Be CRL). To correct for the residual aberrations of the lens, a phase plate was installed immediately following the lens stack. (**a**) For ptychography the test object—an array of Siemens stars—was placed in the vicinity of the focal plane. The sample was scanned transversely to the beam (*x–y* raster scan) and far-field diffraction patterns were recorded at each position. (**b**) Ronchigrams were recorded using a grating test sample with a distinct grating period positioned along the optical axis. Further details can be found in the Methods.

**Figure 2:**
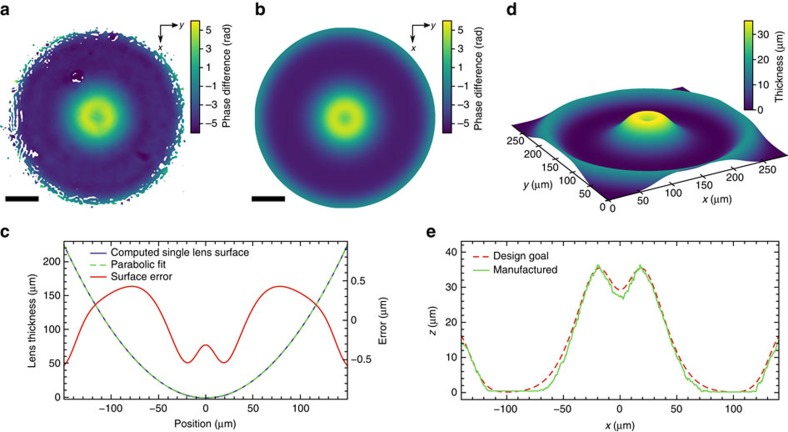
Initial lens characterization and phase plate design. (**a**) Measured wavefront deformation at the lens exit compared to a spherical wave. (**b**) Phase error of a modelled lens stack at the lens exit. Scale bars in **a**,**b** correspond to 50 μm. (**c**) Deformation of every lens surface in the modelled stack of 20 beryllium compound refractive lenses used to generate **b**. The surface error (solid red line) is enhanced by the axis on the right side. (**d**) Model of the SiO_2_ phase plate to correct for errors shown in **a**–**c**. (**e**) Surface profile of the manufactured corrective SiO_2_ phase plate using ultrashort-pulse laser ablation compared with the design goal **d** as measured by a laser scanning microscope.

At the Matter in Extreme Conditions (MEC) endstation^24^ of the LCLS, focused X-ray pulses are, for example, used for magnified phase-contrast imaging of shock waves in matter^18^, to locally probe the structure in shocked matter by diffraction or to generate high-energy-density states in matter. For this purpose, the LCLS beam is focused slightly before or at the sample position, respectively, using refractive X-ray lenses. In our experiment, 20 refractive lenses made of beryllium were used to focus the beam (photon energy: *E*=8.2 keV) with a focal length of ∼250 mm (Fig. 1). It was characterized in detail by ptychography with a test object placed slightly out of focus (Fig. 1a) and showed pronounced spherical aberration^4^ that was also confirmed by the Ronchi test^25^,^26^ (Fig. 1b). By propagating the wavefield back to the exit plane of the lens and subtracting a spherical wave corresponding to the focal distance, a detailed map of the phase errors, which are responsible for the aberrations in the focus, was determined quantitatively (Fig. 2a). The measured wavefront deviation was used to model the shape error of individual lens surfaces (see optics characterization in the Methods). It was found that shape inaccuracies smaller than 500 nm per lens surface (Fig. 2c) reproduce the measured wavefront deviation shown in Fig. 2a very accurately as depicted in Fig. 2b with the standard deviation of 0.3 rad between the two. A reduction of phase errors in individual refractive lenses is technically extremely challenging, as the shape errors for individual lens surfaces lie in the range of the current fabrication tolerances. Therefore, we pursued the following approach: the resulting accumulated phase error of the whole lens stack was corrected for by a single phase plate placed after the stack (Fig. 1). It was made out of an amorphous SiO_2_ substrate by ultrashort-pulse laser ablation^27^. The material was chosen based on its radiation hardness and well-known fabrication parameters for laser micromachining^28^. The phase plate was locally structured in thickness such as to add an additional phase shift that compensates the measured residual phase error of the whole lens (see phase plate design and fabrication in the Methods). The modelled wavefront deformation (Fig. 2b) defines the shape of the phase plate as shown in Fig. 2d,e.

### Diffraction-limited X-ray focusing

**Figure 3:**
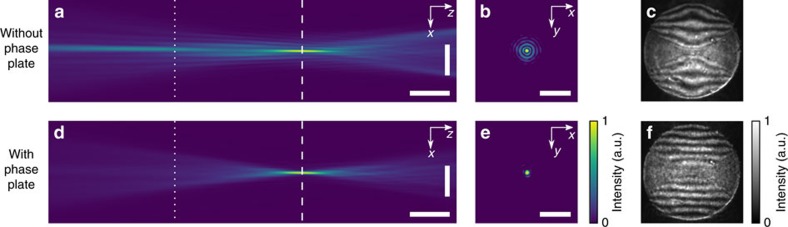
Aberration correction by a corrective phase plate. (**a**) Beam caustic retrieved from the ptychographic reconstruction. Scale bars are 2 μm and 1 mm in *x* and *z* direction, respectively. (**b**) Logarithmic intensity distribution in the focal plane as marked by the dashed line in **a**. Scale bar represents 2 μm in *x* and *y* direction. (**c**) Ronchigram recorded at the dotted position in the beam caustic **a**. Insets **a**–**c** are without the phase plate. (**d**) Beam caustic retrieved from the ptychographic reconstruction. Scale bars identical to **a**. (**e**) Logarithmic intensity distribution in the focal plane as marked by the dashed line in **d**. Scale bar identical to **b**. (**f**) Ronchigram recorded at the dotted position in the beam caustic **d**. Insets **d**–**f** are with the phase plate installed. Insets **a**,**b**,**d**,**e** share the same colour bar as well as **c**,**f**.

By introducing the phase plate as an additional optical component behind the lens stack, the X-ray focus has been significantly improved. The performance of the corrected focusing optics was investigated at the LCLS, and was also confirmed in different experiments at the synchrotron radiation sources PETRA III (DESY, Hamburg, Germany) and Diamond Light Source (DLS) (Oxfordshire, UK). For comparison, the optics were characterized with and without the corrective phase plate by ptychography and the Ronchi test. Figure 3a–c show the results without the corrective phase plate measured at LCLS: the caustic (Fig. 3a) showed a clear signature of spherical aberration, where the paraxial rays are focused to a point upstream of the focus for the peripheral rays. In the focal plane (dashed line in Fig. 3a), defined as the plane with highest peak intensity, the side lobes around the central focal speckle were strongly pronounced (Fig. 3b). The spherical aberration was confirmed by the Ronchi test that showed distorted interference fringes characteristic of spherical aberration (Fig. 3c). The associated root meansquare (r.m.s.) wavefront error in the exit pupil of the optics was 0.23 *λ*.

**Figure 4:**
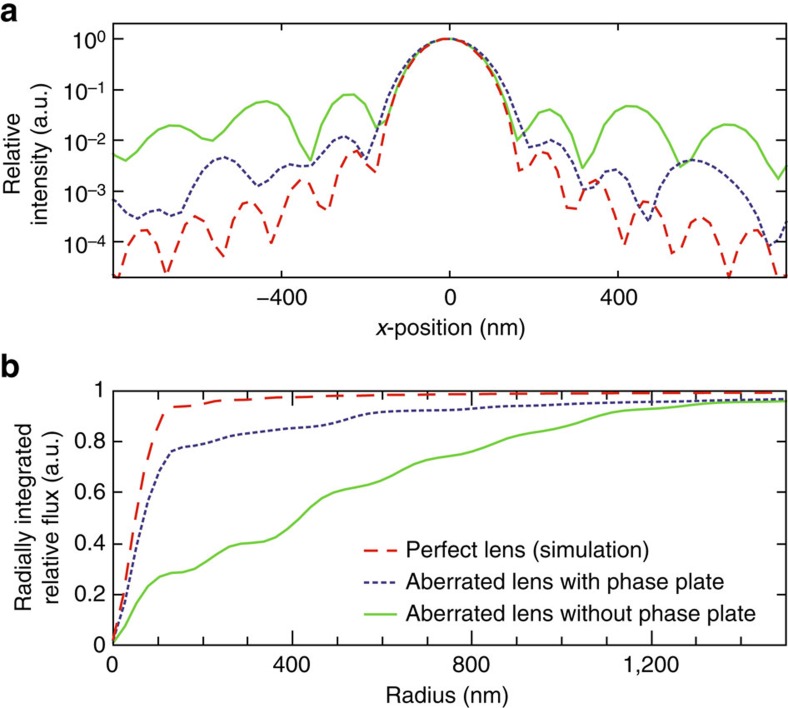
Improved focal spot characteristics. (**a**) Horizontal slice (*x*-direction, logarithmic scale) through the focal plane depicted in Fig. 3b,e and for an ideal lens. (**b**) Radially integrated intensity distribution around the centre of the focal spot. The solid green line represents the results for the uncorrected lens (without the phase plate), the dotted blue line represents the phase plate corrected lens, and the dashed red line represents the modelled aberration-free lens in both **a**,**b**.

Results for the optics with the corrective phase plate obtained at LCLS are shown in Fig. 3d–f: the caustic showed a nearly Gaussian focus (Fig. 3d), and the side lobes in the focal plane were significantly reduced (Fig. 3e). In addition, the Ronchigram showed almost undistorted straight lines (Fig. 3f), which are characteristic of an aberration-free focus. Wavefront errors in the exit pupil were reduced down to 0.06 *λ* r.m.s. The focal properties for these measurements are analysed in more detail in Fig. 4a. The relative intensity of the side lobes decreased by an order of magnitude for the corrected lens (dotted blue line) versus the aberrated one (solid green line). Compared with a perfect focus created by a completely aberration-free lens (dashed red line), the side lobes of the corrected focus were only larger by a factor of two. Figure 4b shows the radially integrated relative flux as a function of position from the optical axis. For the corrected optics, ∼75% of the integral flux was within the central speckle covering an area with a 125 nm radius. On the other hand, the uncorrected focus only concentrated ∼25% of the flux in the central speckle, distributing ∼75% of the radiation over an area with an 800 nm radius. In all experiments the relative flux in the central speckle increased from 0.23(6) to ∼0.80(6) as compared with the focus of an aberration-free ideal lens. For the corrected lens, the Strehl ratio, that is the ratio of the maximal intensity in the focus compared with that of ideal optics, improved from 0.29(7) to 0.87(5) for all experiments and is thus larger than 0.8, above which an optical system is typically considered as diffraction limited^29^. In comparison to that, the lateral full-width at half-maximum beam size was almost unchanged. We determined 156(3) nm and 151(3) nm for the corrected and uncorrected focus, respectively, which is only slightly larger than that of the ideal focus of 143(1) nm.

## Discussion

In conclusion, we successfully demonstrated the correction of residual spherical aberration in a stack of Be CRLs by a corrective phase plate, thus achieving diffraction-limited focusing with a Strehl ratio of 0.87(5). Minimizing the intensity in the outer lobes is not only critical for high power density experiments, but also for scanning microscopy applications, as side lobes may contribute significantly to the measured signals. The approach provides a versatile and general solution to correct aberrations for a variety of X-ray optics ranging from refractive optics as demonstrated here, diffractive optics such as Fresnel zone plates and multilayer Laue lenses, as well as reflective mirror optics. All these optics may be corrected to improve their performance beyond current manufacturing limitations. This effectively solves the problem of aberrations and of technological limitations in the fabrication of optics in the hard X-ray regime. Therefore, X-ray phase plates could become an essential technology for the next generation of XFEL and storage ring sources. Diffraction-limited X-ray foci of nm size are not only crucial to improve existing experiments, but they might also enable novel science opportunities such as nonlinear Compton scattering^30^ or pair creation by photon–photon scattering^31^.

## Methods

### Experimental setup

The experiments were conducted at three different facilities (MEC endstation at the LCLS, beamline P06 at PETRA III and beamline I13-1 at DLS) with similar experimental setups. The broadband X-ray radiation was monochromatized by silicon crystals to a photon energy of *E*=8.2 keV. The flat beam was focused by a set of 20 Be CRLs with a radius of curvature of *R*=50 μm and geometrical aperture of *D*=300 μm. The aperture was defined by a pinhole of *D*=300 μm diameter that matches the geometrical aperture at the entrance of the optics. The phase plate was mounted directly behind the CRL, either in a self-centring special mount or in a motorized mount adjustable in *x* and *y* to investigate tolerable lateral misalignment and to optimize the centring of the phase plate. For the ptychographic scans, an array of small Siemens stars with 50 nm smallest features structured in 1 μm thick tungsten on a diamond substrate was placed into the vicinity of the focal plane (Fig. 1a). The far-field diffraction patterns were recorded using an area detector placed several metres behind the test object (LCLS: in-air CS-PAD 140k at a distance 4.8 m downstream of the sample, DLS: Medipix-3-based Merlin detector at 2.6 m downstream of the sample, PETRA III: Medipix-3-based LAMBDA (Large Area Medipix-Based Detector Array) detector at 2.2 m downstream of the sample). For the Ronchi test (Fig. 1b) we used a high-resolution X-ray camera magnifying a scintillator screen and gratings with 270 nm period structured into 1 μm thick tungsten on a diamond substrate.

### Optics characterization

The recorded far-field diffraction patterns were processed with a ptychographic algorithm (ePIE)^32^ with position refinement^4^ to reconstruct both the complex object transmission function of the test sample, as well as the complex-valued wavefield *Ψ* that illuminated the sample. From the illumination and using the Fresnel–Kirchhoff diffraction integral^33^, the complex wavefield can be calculated at any point along the optical axis, creating a beam caustic as shown in Fig. 3. We propagated *Ψ* back to the lens exit to obtain *Ψ*_exit_(*x*, *y*). To reveal phase errors, we subtracted the phase of a spherical wave 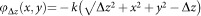 with radius Δ*z*=*z*_*f*_−*z*_exit_ that was matched to the distance from lens exit *z*_exit_ to focal plane *z*_*f*_ of the Be CRL^34^:


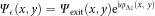


Residual deviations from a plane phase represent the aberrations present in the given optics (Fig. 2a) and can be evaluated to accurately model their shape error (Fig. 2c) and to design corrective elements like the phase plate characterized in Fig. 2d,e. To model the error of a single lens surface, we propagated a plane wave from lens to lens through the stack, treating every lens as thin. Under the assumption of an identical deformation for every lens and radial symmetry, the shape was iteratively refined until the total phase deviation shown in Fig. 2b matched the measured one in Fig. 2a (*σ*≈0.3 rad).

In addition, an independent Ronchi test was carried out as depicted in Fig. 1b. Here, a diffraction grating was placed in the vicinity of the focal plane. The grating period was chosen so that the diffracted beam cones overlap each other roughly half way (see Fig. 1b and Ronchigrams in Fig. 3c,f). The interfering amplitudes from the cones created a distinct fringe pattern. Straight interference fringes indicate an aberration-free system (Fig. 3f), and bent lines can reveal coma, astigmatism or, in our case, spherical aberration (Fig. 3c) on a single-shot basis^25^. Thus, Ronchi patterns were used to align the lens and the phase plate to one another.

### Phase plate design and fabrication

From the reconstructed wavefront deviation 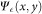 a phase plate shape *z*_PP_(*x*, *y*) can be designed, so that the induced phase shift in the material 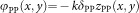 compensates any present wavefront deviations:


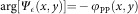


The phase plate structure was created in an amorphous SiO_2_ substrate (Vitreosil 077, *ρ*=2.2 g cm^−3^) with a thickness of 118(2) μm.

We used an ultrashort-pulsed laser system (Trumpf TruMicro 5050) to enable nonlinear absorption in the focal region for highly localized energy transfer^27^,^28^. The laser emits pulses with durations of 8 ps at a wavelength of 1030, nm. The pulse energy was set to 0.2 μJ to facilitate precise ablation of the desired structure. Furthermore, a microscope objective with a NA of 0.4 served to focus the pulses onto the substrate, providing a spot size of ∼1 μm. The sample was moved underneath the focus using a nanopositioning system. Based on the computed surface profile of the phase plate, individual layers with a thickness of ∼1 μm were sequentially ablated.

The phase plate has a transmission of ∼54%. It can be increased up to 95% if the thickness of the supporting SiO_2_ substrate is minimized, yielding diffraction-limited and highly efficient X-ray optics. The system is applicable over a large photon energy range when correcting refractive optics, as the refractive power is nearly material independent far from absorption edges. Thus, a single phase plate can correct aberrations of a refractive lens over a large photon energy range.

### Data availability

Data supporting the findings of this study are available from the corresponding author on request.

## Additional information

**How to cite this article:** Seiboth, F. *et al*. Perfect X-ray focusing via fitting corrective glasses to aberrated optics. *Nat. Commun.* **8**, 14623 doi: 10.1038/ncomms14623 (2017).

**Publisher's note**: Springer Nature remains neutral with regard to jurisdictional claims in published maps and institutional affiliations.

## Supplementary Material

Peer Review File
