# Supplementary material for: Perfect X-ray focusing via fitting corrective glasses to aberrated optics
Source: Nat Commun. 2017 Mar 1;8:14623. doi: 10.1038/ncomms14623 (PMC5337966; doi:10.1038/ncomms14623)
Supplement: Peer Review File [file ncomms14623-s1.pdf]

Editorial Note: this manuscript has been previously reviewed at another journal that is not operating a transparent peer review scheme. This document only contains reviewer comments and rebuttal letters for versions considered at Nature Communications.

REVIEWERS' COMMENTS:

Reviewer #1 (Remarks to the Author):

The paper of Seiboth et al. reports the correction of residual spherical aberrations in a compound refractive lens by a corrective phase plate. They measured the residual aberrations using ptychography in order to manufacture the phase plate and realized a diffraction-limited X-ray focusing with a Strehl ratio above 0.8. The paper is well written and the data and results are clearly presented. Overall, I think that Nature Communications is an appropriate journal for this work.
